# Virtual staining for pixel-wise and quantitative analysis of single cell images

**DOI:** 10.1038/s41598-023-45150-y

**Published:** 2023-11-06

**Authors:** Abdurrahim Yilmaz, Tuelay Aydin, Rahmetullah Varol

**Affiliations:** 1 Universität der Bundeswehr München, 85579 Neubiberg, Germany; 2 Present Address: Imperial College London, London, SW7 2BX United Kingdom

**Keywords:** Cellular imaging, Machine learning

## Abstract

Immunocytochemical staining of microorganisms and cells has long been a popular method for examining their specific subcellular structures in greater detail. Recently, generative networks have emerged as an alternative to traditional immunostaining techniques. These networks infer fluorescence signatures from various imaging modalities and then virtually apply staining to the images in a digital environment. In numerous studies, virtual staining models have been trained on histopathology slides or intricate subcellular structures to enhance their accuracy and applicability. Despite the advancements in virtual staining technology, utilizing this method for quantitative analysis of microscopic images still poses a significant challenge. To address this issue, we propose a straightforward and automated approach for pixel-wise image-to-image translation. Our primary objective in this research is to leverage advanced virtual staining techniques to accurately measure the DNA fragmentation index in unstained sperm images. This not only offers a non-invasive approach to gauging sperm quality, but also paves the way for streamlined and efficient analyses without the constraints and potential biases introduced by traditional staining processes. This novel approach takes into account the limitations of conventional techniques and incorporates improvements to bolster the reliability of the virtual staining process. To further refine the results, we discuss various denoising techniques that can be employed to reduce the impact of background noise on the digital images. Additionally, we present a pixel-wise image matching algorithm designed to minimize the error caused by background noise and to prevent the introduction of bias into the analysis. By combining these approaches, we aim to develop a more effective and reliable method for quantitative analysis of virtually stained microscopic images, ultimately enhancing the study of microorganisms and cells at the subcellular level.

## Introduction

Immunochemical staining refers to a technique used to enhance the visibility of cells and other biological structures under the microscope^1^. Different biomarkers (dyes) are used for labeling biological samples depending on the structure being observed. These dyes attach to designated areas on the target structure and, when exposed to a certain wavelength of light, they emit light at a distinct wavelength. Thus, staining allows one to see the details of particular structures that are normally invisible due to optical limits. Generative deep neural networks such as generative adversarial networks (GANs) and autoencoders (AEs) can be used to learn a mapping from other imaging modalities to fluorescent images of specific subcellular structures. Such models are valuable in biological applications as they eliminate the need for labeling.

To stain these structures, various systems such as fluorescence microscopy, specific dyes for the targeted structure, and different staining protocols are required^2,3^. However, use of dyes on biological samples damages their integrity and diminishes their viability^4^. Furthermore, the diffusion of the dye may be at varying levels, which may impair visibility, because the staining method is not standardized^5^. The margin of error grows larger in research where quantitative analysis is important. Immunostaining has several potential sources of inaccuracy, such as the background noise present in a fluorescence microscope^6^, non-specific binding of the antibodies^7^, variability in staining intensity due to experimental conditions^8^, and the potential for photobleaching of fluorescent dyes, all of which must be considered and addressed^9^.

There are many augmented microscopy studies for microorganisms and biological cells^10^. The field of augmented microscopy has witnessed notable progress in recent times, leading to the emergence of novel solutions. However, a thorough comprehension of the wider ramifications of these breakthroughs still eludes researchers. Although numerous studies provide generic approaches, there is a noticeable lack of research that specifically focuses on applications. In addition, the discipline would derive advantages from the implementation of more rigorous quantitative studies. These analyses should encompass a comparison of virtually stained samples and their conventionally stained equivalents, as well as an examination of generating images. It is worth noting that there is a limited body of research that extensively examines the quantitative evaluation of factors that hold clinical significance. With generative networks, methods such as data augmentation for cell images were presented^11^. First multichannel virtual staining of cell structures was performed using conditional AEs^12^. Multiple cell types and imaging techniques were used for virtual staining applications^13^. A generative network was used to develop a probabilistic model that can predict specific fluorescent protein localizations from cellular and nuclear morphology^14^. Using a transmitted light microscope, bright-field images (BFIs) were collected from different sections of the cell, and then virtually stained in 3D^15^. GAN models were also used for virtual staining of histopathology slides using label-free confocal images without the use of dyes^16^. Lastly, 3D image-to-3D image translation of subcellular structures of cells was stained using a variational AE-based approach^2^.

The generative networks for virtual staining have previously been used for either virtual staining of histopathological specimens^17^ or subcellular structures^18^. Studies have mostly focused on inpainting applications^19–21^. Virtual staining can also be used for quantitative analysis of immunochemically stained samples for particular applications where the underlying molecular structure is encoded in the morphological features of the sample^18,22^. Therefore, generative networks can be used in place of a fluorescence microscope and dyes to develop pre-trained models which can be used for various applications such as cell counting, cell viability evaluation, and drug screening. Furthermore, generative networks suppress variations that arise from incorrect measurements, as they learn a statistical distribution of the output space when provided with sufficient training samples^23^. Quantitative analysis based on immunostaining can be performed to calculate parameters such as the DNA fragmentation index (DFI), which quantifies the proportion of sperm cells with fragmented DNA, serving as an indicator of sperm quality and integrity.

We present a virtual staining based quantitative analysis methodology that can be used to predict the underlying molecular structure such as the localisation of subcellular structures or proteins from a brightfield image. The proposed method is useful in cases where the molecular structure is somehow encoded in the morphological structure of the sample. We tested the proposed methodology using the human sperm dataset which was introduced by McCallum et al. in a study where they trained a regressive convolutional neural network (CNN) model to directly predict DFI values of single sperm cells from brightfield images^24^. We refer to this study as “the original study” throughout the manuscript. Our objective was to perform a quantitative analysis of a human sperm dataset by calculating DFI. We then compared our results with those of the original study and the ground-truth values. This simple pipeline can facilitate the application of virtual staining studies in well-known immunostaining scenarios, aiming to enhance daily laboratory practices. The proposed methodology could be used to obtain molecular information without staining for particular cases where the morphological information is largely related to the desired molecular information. Thus, the toxicity introduced by the staining process would be eliminated and the sample can be investigated for a long period. Even though generative networks were previously used for virtual immunostaining, to the best of our knowledge, this is the first application where the DNA-fragmentation index of sperm cells was calculated through virtual staining of their brightfield images.

## Results

DFI values were computed using the output of GAN models. To assess the performance of these GAN models, we calculated the mean absolute error (MAE) and mean absolute percentage error values for the DFI value of ground-truth and generated images. These values were determined using the following equations:

1 $$\begin{aligned} \text {MAE}= & {} \sum _{i=1}^{\text {N}}\frac{|y_i-x_i|}{\text {N}} \end{aligned}$$

2 $$\begin{aligned} \text {MAPE}= & {} \frac{1}{\text {N}} \sum _{i=1}^{\text {N}}\frac{|y_i-x_i|}{y_i} \end{aligned}$$

We individually calculated the MAE and MAPE metrics for all donors. The MAE and MAPE values of virtual staining for both the training and testing phases are displayed in Table 1. The generative network, based on the pix2pix architecture, produced an MAE value of 0.0204±0.0033 for the training dataset and 0.0220 for the test dataset. The crucial takeaway is that these results demonstrate the feasibility of obtaining reliable quantitative outcomes from virtually generated immunostaining images. To increase the stability of the training process we also compared the results with another architecture, pix2pix++, with a spectral normalization layer incorporated into the discriminator network. The results for this network are also given in Table 1 with a mean MAE value of 0.0199. These results are also important to demonstrate the repeatability of the proposed method.

**Table 1:** Mean absolute error and mean absolute percentage error values of virtually stained images for training and test datasets and for the original study.

	pix2pix (noisy)		pix2pix (denoised)		pix2pix++ (denoised)	
	MAE value	MAPE value	MAE value	MAPE value	MAE value	MAPE value
Donor 1—test	0.0235	0.2403	0.0220	0.2250	0.0198	0.2025
Donor 2	0.0243	0.3868	0.0179	0.2857	0.0164	0.2611
Donor 3	0.0229	0.1461	0.0257	0.1643	0.0230	0.1467
Donor 4	0.0211	0.1946	0.0191	0.1761	0.0215	0.1981
Donor 5	0.0217	0.3012	0.0179	0.2482	0.0182	0.2521
Donor 6	0.0229	0.2417	0.0214	0.2250	0.0206	0.2165
Mean	0.0215	0.2517	0.0207	0.2207	0.0199	0.2114
S.D.	0.0022	0.0840	0.0027	0.0451	0.0023	0.0349
Mean of original study			0.023			
p-value pix2pix noisy and denoised			0.15			
p-value pix2pix noisy and pix2pix++			0.03			
p-value pix2pix denoised and pix2pix++			0.64			

It is important to note that the original paper presents saliency maps of the neural network. Image regression from BFI to calculate the DFI value can cause neural networks to learn incorrect features and potentially create bias in the calculations. Specifically, image regression models may take into account regions outside of the sperm head, such as the background or the tail of the sperm, as they do not inherently know where to focus their attention. As a result, a method that generates output using only the relevant region for quantitative analysis is necessary.

## Discussion

In this paper, we present a comprehensive pipeline for virtual staining-based quantitative analysis. Virtual staining is a technique where conventional histological staining is replaced with computational methods to produce similar or better results. This method eliminates the need for the expensive and time-consuming process of preparing and staining tissue samples, making it more efficient and cost-effective.

Furthermore, we demonstrated a denoising technique for fluorescent images, which is crucial for obtaining accurate quantitative results. By removing noise from the images, the authors were able to calculate DFI values more accurately.

To generate dsDNA and ssDNA fluorescent images, we trained a generative model using a human sperm dataset. This model can be used for other applications, such as clinical and laboratory immunostaining problems particularly for applications where the underlying molecular structure can be predicted from morphological features. We also compared the DFI values calculated using the generated images to the ground-truth DFI values, demonstrating the effectiveness of the proposed approach. The proposed methodology could be used to obtain molecular information without staining for particular cases where the morphological information is largely related to the desired molecular information. Thus, the toxicity introduced by the staining process would be eliminated and the sample can be investigated for a long period.

**Figure 1:**
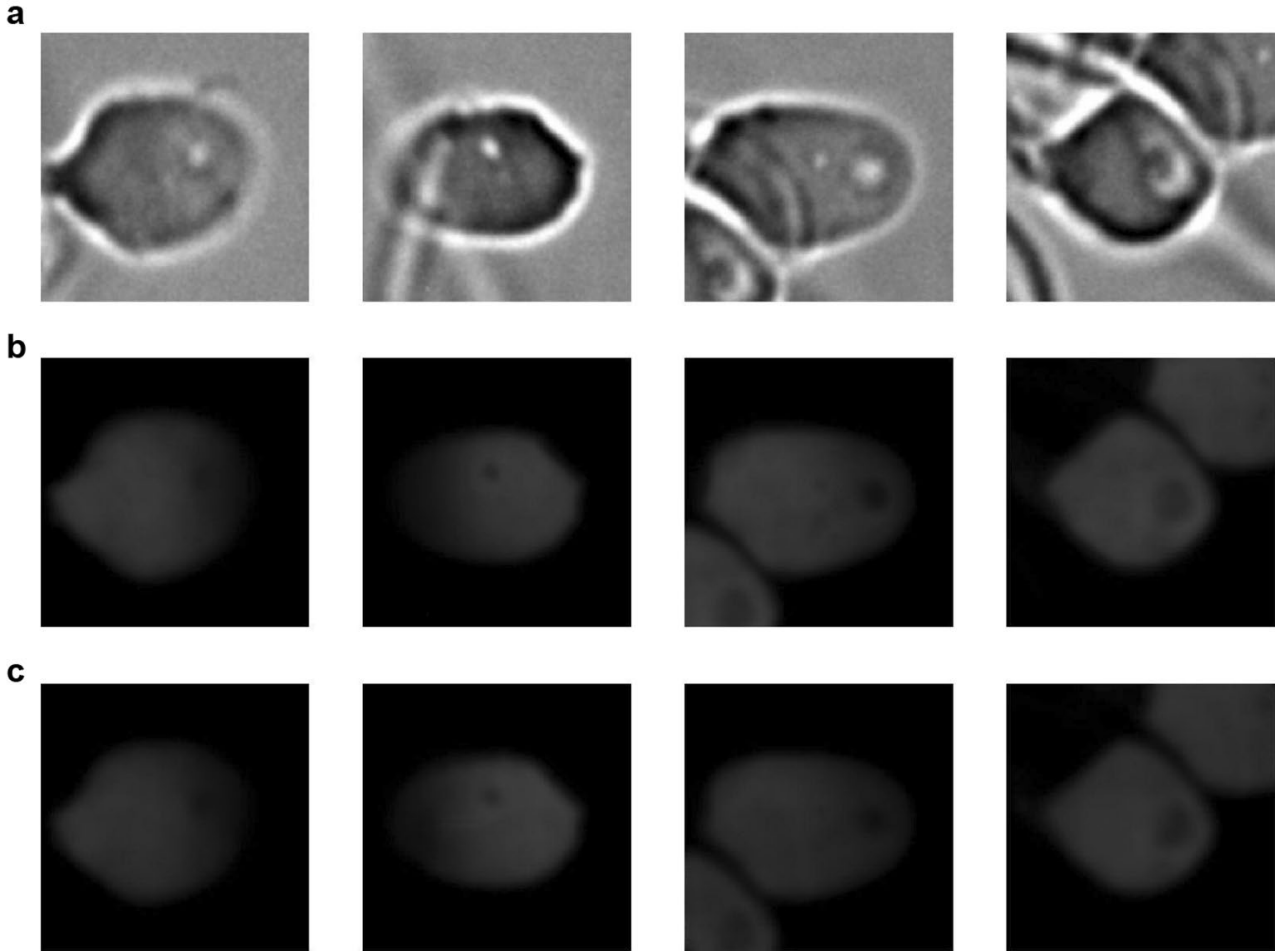
Shows the example images for this study. Generative networks can correctly predict intensity values. (**a**) Source bright-field images, (**b**) Source dsDNA fluorescence images, and (**c**) Generated dsDNA fluorescence images.

At this juncture, generative networks offer several advantages, including noise reduction and pixel-wise calculation capabilities. Our GAN model can establish relationships between the sperm head area and dsDNA and ssDNA fluorescence images (FI) on a pixel-wise basis. Examples of the generated images are displayed in Fig. 1. For all generated patches, the background noise intensity was determined to be zero. Consequently, the GAN model learned only the intensity values for the sperm head region due to its conditional structure. This inherent feature of GANs enables the automated quantification of DFI values.

Furthermore, our GAN model exhibits robustness against artifacts, such as lens distortions, and can accurately predict challenging regions, including the edges of cells. This adaptability makes the GAN model an effective tool for generating virtual staining images that can be reliably analyzed quantitatively.

In the future, pre-trained models can be developed for common clinical and laboratory immunostaining problems, which will make the analysis more efficient and accessible for clinicians. Additionally, the creation and distribution of software for use in cloud-based online platforms and applications like ImageJ will also benefit researchers and clinicians. It is noteworthy to mention that we utilized brightfield images of stained samples rather than truly unstained specimens. This aspect can be improved upon in the future by constructing an imaging system that can take images of sperm cells before and after staining process and match individual cells in these images.

Different imaging techniques may provide higher precision quantitative analysis, especially for the investigation of DFI^22^. Therefore, further studies can be conducted to explore the potential of different imaging techniques for quantitative analysis. In addition to this, numerous models have been previously created in the field of virtual staining, with a primary emphasis on quantitative analysis. Nevertheless, it is essential to recognize the constraints of current methodologies, particularly their limited adaptability for direct application in a clinical setting. While our solution offers a promising direction with a customized model tailored for potential use in clinical applications, it requires further validation and refinement. In the future, with rigorous testing and optimization, our method holds the potential to be seamlessly incorporated into targeted clinical applications, bridging the gap that currently exists. This technological progress not only serves to connect the divide between theoretical analysis and actual implementation, but also lays the foundation for the development of virtual staining techniques that are more applicable in clinical settings in the coming years. The recognition of previous research contributions is essential. However, our study offers a notable advancement in the application of virtual staining techniques to provide practical advantages in clinical settings.

## Methods

**Figure 2:**
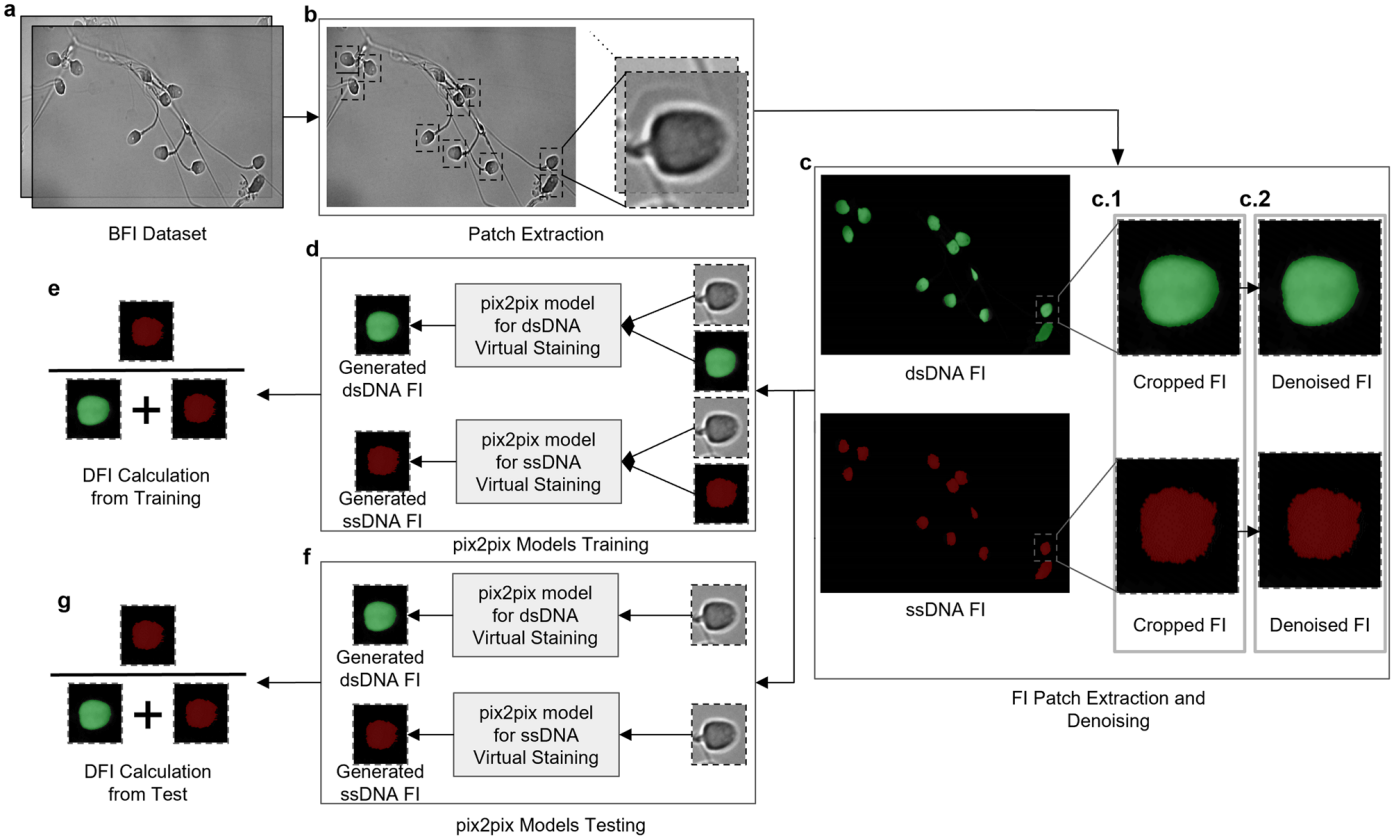
Workflow. (**a**–**b**) First, the exact position of bright-field patches of sperm cells was determined. (**c.1**) The fluorescence patches were extracted based on the bright-field patch positions. (**c.2**) Denoising was applied to improve success of generative networks. (**d**) Two separate generative adversarial networks based on pix2pix architecture were trained both dsDNA staining and ssDNA staining. (**e**) DFI values for each sperm cells were calculated automatically using intensity values of generated images. (**f**) Trained generative adversarial networks were tested using bright-field patches. (**g**) DFI values for each sperm cells were calculated to measure success of virtual staining models.

In this study, we generated a virtual staining dataset using human sperm dataset^24^. Then, we denoised fluorescence images (FI), trained two generative networks, and compared with original study as shown in Fig. 2. The application was developed in Python. OpenCV was used for feature detection, scikit-image for image resizing, and Keras for training deep neural networks.

### Human sperm cell dataset

Acridine orange test is used to observe DNA integrity of sperm cells. This test generates green and red fluorescence to tag double and single stranded DNA (dsDNA, ssDNA). Here, we used a human sperm cell dataset that includes 274 BFIs, and their green and red fluorescence counterparts from 6 donors^24^. By using this dataset, we extracted matching sperm head patches for each modality. We excluded Donor 1 from training dataset and used it for testing the trained model. The virtual staining dataset of Donor 2-6 for training consists of 555 BFI, dsDNA and ssDNA FI patches with size of 256 px $\times$ 256 px. Here the critical parameters is the number of individual sperm cells since the DFI value varies greatly for sperm cells that are taken from the same donor and the important task is to distinguish high and low quality sperm cells for each individual.

### Feature detection

To generate a pixel-wise virtual staining dataset, we calculated the homography matrices to match each modality with each other. First, we used Scale-Invariant Feature Transform (SIFT) to extract the visual features that we can use for keypoint matching. SIFT features were used because they are generally robust to noise and occlusion. Then, we used Fast Library for Approximate Nearest Neighbors (FLANN) based descriptor matcher with two best matches (k-nearest neighbor matching). Matched descriptors were filtered using the Lowe’s ratio test to filter out unreliable matches. After finding the positions of descriptors in the BFI, a homography matrix based on RANdom SAmple Consensus (RANSAC) was calculated to obtain corresponding pixel position. The RANSAC algorithm is effective at calculating a homography matrix even in the presence of outliers or mismatches in the point correspondences, as it focuses on the most consistent subset of correspondences. Using the homography matrix, perspective transformation was applied, and corresponding patches from each modality were cropped. Finally, all patches were resized to 256 px $\times$ 256 px using bilinear scaling.

### Denoising

**Figure 3:**
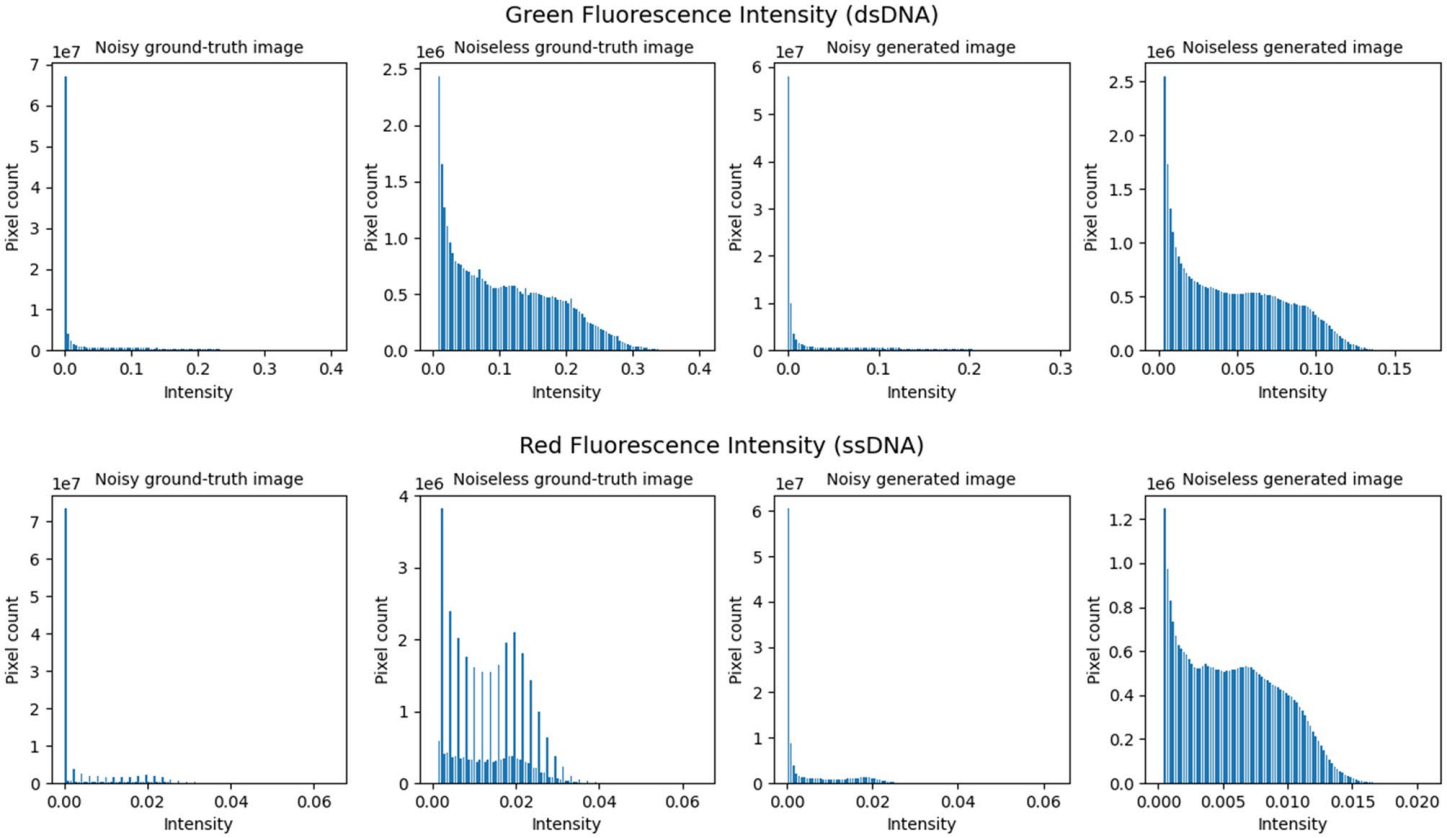
Shows the histograms of noisy and noiseless ground-truth and generated images for both green and red fluorescence images. The outstanding line in histograms of noisy images represents background pixels. Its value is accepted to be the background noise value.

When performing quantitative analysis, it is crucial to minimize errors to obtain accurate results. However, FIs can be affected by various sources of noise that can introduce bias or errors into quantitative calculations. To obtain correct DFI values, we removed noise from FIs. This was achieved by calculating a local noise value for each dsDNA FI patch based on its background signal value. To do this, we stacked all intensity values for a patch in an array and created a histogram using these values. The mean of the most frequently occurring three values was assumed to be the noise value for the patch. This noise value was then subtracted from the dsDNA patch. Additionally, the positions of the pixels equal to one of the most frequently occurring three values were saved for noise calculation of the ssDNA patch. The mean intensity value of these pixels for the corresponding ssDNA FI patch was used as the noise value for the ssDNA patch, which was then subtracted from the patch. Finally, we calculated DFI values for each sperm cell automatically using the intensity values of both dsDNA and ssDNA. The histograms for noisy and denoised images are shown in Fig. 3. In these histograms, the zero valued pixels are not counted. It can be seen that the low intensity background noise was successfully removed. Our objective behind denoising was to eliminate consistent background noise which might interfere with our model’s learning and subsequent image generation. We selected the three most frequently occurring pixel values in the background based on their prevalence, and by subtracting these values, we aimed to uniformly suppress the pervasive background noise across the images. The same pixels were used for denoising both dsDNA and ssDNA images since we observed that the noise patterns were consistent across both channels.

### Generative networks for virtual staining

After preparing a suitable dataset, generative networks such as GANs and AEs can be employed for image-to-image translation tasks. In this study, we utilized a paired image translation architecture, specifically the pix2pix model, to generate dsDNA and ssDNA patches. The total loss function for the pix2pix model is given by:

3 $$\begin{aligned} L_{pix2pix} = \lambda _{L1} L_{L1} + \lambda _{GAN} L_{GAN} \end{aligned}$$

where $L_{L1}$ represents the L1 loss between the generated image and the target image, and $L_{GAN}$ denotes the adversarial loss that measures the discriminator’s ability to distinguish between the generated image and the target image. $\lambda _{L1}$ and $\lambda _{GAN}$ are hyperparameters that control the weight of each respective loss term. One key advantage of using the pix2pix model is that it enables pixel-level image-to-image translation while requiring only a small amount of training data compared to many contemporary studies in the field that utilize larger datasets^2,16,22^.

In our approach, we provided the network with BFI patches and denoised dsDNA or ssDNA patches as input. Prior to training, we scaled the pixel values of all images from the range [0,255] to [-1,1]. The generator was defined using seven encoder blocks with Leaky Rectified Linear Unit (LeakyReLU) activation function (with the number of filters set to 64, 128, 256, 512, 512, 512, 512); one bottleneck layer with ReLU activation function and 512 filters; seven decoder blocks with skip connections and ReLU activation function (number of filters being 512, 512, 512, 512, 256, 128, 64); and a hyperbolic tangent (tanh) function as the final layer activation function of the generator.

**Figure 4:**
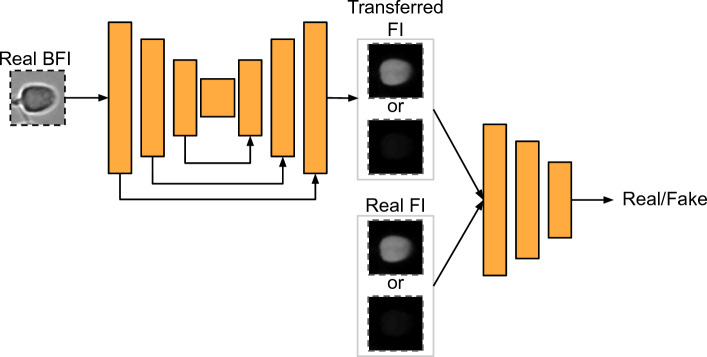
Shows the pix2pix architecture. The generative adversarial network accepts source bright-field images and source fluorescence images as input and generates virtually stained fluorescence images.

The discriminator was defined using five convolutional layers with ReLU activation function (number of filters set to 64, 128, 256, 512, 512); and a sigmoid function as the final layer activation function. The GAN network architecture is depicted in Fig. 4. We trained two separate models for the two FI modalities using binary cross-entropy and mean absolute error (MAE) losses, and the Adam optimizer on an RTX 3090 GPU for a total of 1000 epochs.

Subsequently, two virtually stained images (dsDNA and ssDNA) for each sperm head were generated using these GAN models. Finally, all generated images were rescaled from the range [-1,1] back to [0,1] in order to perform DFI calculations. DNA fragmentation refers to the breaking of DNA strands, which can be assessed by differentiating between double-stranded DNA (dsDNA) and single-stranded DNA (ssDNA) images. dsDNA images show DNA in its native, intact form, while ssDNA images reveal the presence of fragmented DNA regions. The DNA fragmentation index (DFI) is a measure used to quantify the degree of fragmentation. It is calculated as:

4 $$\begin{aligned} \text {DFI} = \left( \frac{\text {Area of ssDNA}}{\text {Area of ssDNA} + \text {Area of dsDNA}} \right) \times 100\% \end{aligned}$$

This approach allowed us to generate high-quality virtual staining images that could be used for further quantitative analysis (Fig. 1).

### pix2pix++ architecture

Training of GANs is recognized for its instability, particularly in the early stages of the training process^25^. The generator and discriminator can engage in a “cat-and-mouse” dynamic, each striving to outperform the other. This instability renders the training process highly sensitive to hyperparameter settings, and achieving convergence can be difficult. To address this instability issue, one effective approach is to implement spectral normalization in every layer of the model.

Spectral normalization is a technique that constrains the Lipschitz constant of the discriminator function by normalizing the spectral norm of each layer’s weight matrix. This technique has been shown to stabilize the training process and improve the quality of generated images^26^. The introduction of spectral normalization has been empirically observed to improve training stability by controlling the exploding and vanishing gradient during training^27^. To improve upon the results obtained from the pix2pix architecture, we used the pix2pix++ architecture, which is a modified version of the pix2pix model that incorporates spectral normalization in the discriminator network^28^.

The key idea behind spectral normalization is to enforce a Lipschitz constraint on the discriminator’s weight matrices by normalizing their spectral norm. The spectral norm of a matrix is the maximum singular value of that matrix. By constraining the spectral norm, the discriminator’s weights are scaled such that the output Lipschitz constant is at most one. During training, after each discriminator update step, spectral normalization is applied to the discriminator’s weight matrices. This is done by dividing each weight matrix by its spectral norm, ensuring that the Lipschitz constant is within the desired range. The normalization is typically applied using power iteration, which approximates the spectral norm by iteratively applying the weight matrix to a random vector^26^.

## Data Availability

The dataset used in this study was obtained from^24^. The dataset is freely available at https://figshare.com/articles/Deep_learning-based_selection_of_human_sperm_with_high_DNA_integrity/8124932.
