# Author Correction: Virtual staining for pixel-wise and quantitative analysis of single cell images

**DOI:** 10.1038/s41598-023-49320-w

**Published:** 2023-12-15

**Authors:** Abdurrahim Yilmaz, Tuelay Aydin, Rahmetullah Varol

**Affiliations:** 1 Universität der Bundeswehr München, 85579 Neubiberg, Germany; 2 Present Address: Imperial College London, London, SW7 2BX United Kingdom

Correction to: *Scientific Reports* 10.1038/s41598-023-45150-y, published online 06 November 2023

The original PDF version of this Article contained an error in the Author contribution section.

A.Y., T.A and R.V. designed the study, A.Y. trained the networks A.A. and B.A. conducted the experiment(s), C.A. and D.A. analysed the results. All authors reviewed the manuscript.

Now reads

A.Y., T.A., and R.V. designed the study, A.Y. and R.V. trained the networks, A.Y. and R.V. conducted the experiment(s), and A.Y. and R.V. analysed the results. All authors reviewed the manuscript.

The original Article has been corrected.

